# Ultra-broadband directional thermal emission

**DOI:** 10.1515/nanoph-2023-0742

**Published:** 2024-01-16

**Authors:** Qiuyu Wang, Tianji Liu, Longnan Li, Chen Huang, Jiawei Wang, Meng Xiao, Yang Li, Wei Li

**Affiliations:** GPL Photonics Laboratory, State Key Laboratory of Luminescence and Applications, Changchun Institute of Optics, Fine Mechanics and Physics, Chinese Academy of Sciences, Changchun 130033, China; University of Chinese Academy of Sciences, Beijing 100039, China; Key Laboratory of Artificial Micro- and Nano-Structures of Ministry of Education and School of Physics and Technology, Wuhan University, Wuhan 430072, China; State Key Laboratory of Precision Measurement Technology and Instruments, Department of Precision Instrument, Tsinghua University, Beijing, 100084, China

**Keywords:** broadband directional thermal emission, effective medium theory, metamaterial

## Abstract

Directional control of thermal emission over its broad wavelength range is a fundamental challenge. Gradient epsilon-near-zero (ENZ) material supporting Berreman mode has been proposed as a promising approach. However, the bandwidth is still inherently limited due to the availability of ENZ materials covering a broad bandwidth and additional undesired omnidirectional modes in multilayer stacking with increased thickness. Here, we show that broadband directional thermal emission can be realized beyond the previously considered epsilon-near-zero and Berreman mode region. We then establish a universal approach based on effective medium theory to realizing ultra-broadband directional thermal emitter. We numerically demonstrate strong (emissivity >0.8) directional (80 ± 5°) thermal emission covering the entire thermal emission wavelength range (5–30 μm) by using only two materials. This approach offers a new capability for manipulating thermal emission with potential applications in high-efficiency information encryption, energy collection and utilization, thermal camouflaging, and infrared detection.

## 1 Introduction

Thermal emission is ubiquitous in nature. All objects at nonzero temperatures emit or absorb thermal radiation according to fundamental principles of statistical mechanics [1], [2]. The ability to control thermal emission [3], [4], [5], [6] has important and far-reaching impacts on various applications such as radiative cooling [7], [8], [9], [10], thermophotovoltaics [11], [12], [13], thermal camouflage [14], [15], [16], and gas sensing [17], [18]. Conventional thermal emission is typically near-isotropic due to the thermally induced random currents that are spatially and temporally incoherent [5], [19]. Surplus emission in unwanted directions, however, can result in the waste of energy and low efficiency of thermal devices [20], [21]. Therefore, the ability to control the direction of thermal emission is fundamentally important and highly desirable.

**Figure 1:**
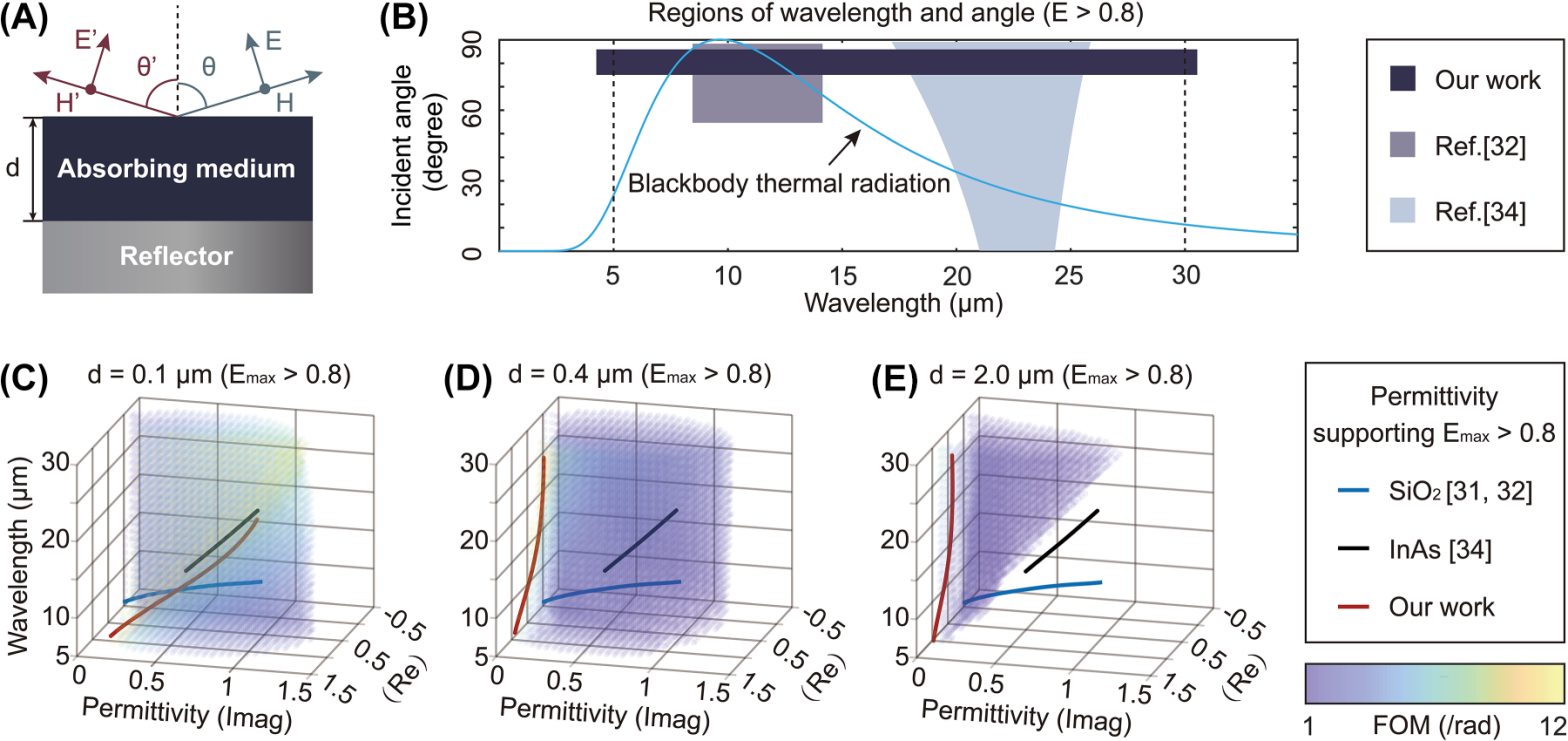
Existing broadband directional thermal emitters versus our work. (A) Schematic of a typical directional thermal emitter consisting of an absorbing medium on top of a metallic reflector. The absorbing medium can be monolayer or multilayer. (B) Comparison of high (>0.8) emissivity wavelength and angular range for previous broadband directional thermal emitters and our work. Our emitter shows directional emission covering the entire thermal emission wavelength range (5–30 μm), as indicated by the blackbody thermal radiation at 300 K (blue curve), as well as a better directionality. The maximum emissivity of the BDTE achieved by Ref. [31] did not exceed 0.8, so Ref. [31] is not shown in (B). (C–E) When the thickness of the top absorbing medium *d* = 0.1 μm, the curves represent the wavelength-dependent permittivity of typical ENZ materials (blue curves: SiO_2_ [31], [32], black curves: InAs [34]) used in the corresponding references as one layer of the absorbing medium with *d* = 0.1 (C), 0.4 (D), and 2 μm (E). Here, we only select the permittivity in the wavelength ranges that support a maximum emissivity greater than 0.8 (*E*_max_ > 0.8) for different thicknesses. For comparison, the permittivity of the top absorbing medium that supports *E*_max_ > 0.8 and FOM between 1 and 12/rad (as indicated by the clusters of colored points) for the TM polarization shows a much broader range. The red curves represent the effective permittivity of the metamaterials (red curves) with appropriate structure parameters demonstrated in this work supporting directional thermal emission over ultra-broad wavelength ranges.

To date, considerable progress has been achieved in directional thermal emission control, using surface phonon polariton structures [22], [23], surface plasmon polariton structures [24], [25], photonic crystals [26], [27], [28], and metasurfaces [29], [30]. These approaches obtained anomalous angular control of thermal emission but were intrinsically restricted to narrowband responses due to their resonant nature. Lately, emitters based on gradient epsilon-near-zero (ENZ) material on top of metal reflector (Figure 1A) have been proposed to overcome this constraint [[Bibr j_nanoph-2023-0742_ref_031] [Bibr j_nanoph-2023-0742_ref_032] [Bibr j_nanoph-2023-0742_ref_033] [Bibr j_nanoph-2023-0742_ref_034]]. In the ENZ region where the real part of the permittivity approaches zero, the emitter supports Berreman mode, a leaky transverse-magnetic (TM) mode that can couple to propagating modes in free space at a range of incident angles [36], [37], [38], [39]. By multilayer stacking of ENZ material, directional emission (maximum emissivity near 0.8) for the TM polarization over the wavelength range of 8–11 μm or 10.5–14 μm has been demonstrated [31]. Followed by this pioneering work, strong (maximum emissivity >0.8) broadband directional thermal emission (BDTE) based on gradient ENZ behavior has been realized based on multilayer polaritonic oxides [32], doped semiconductors [33], [34], [35], and Weyl semimetals [33] with a maximal bandwidth of 9 μm (17–26 μm) [34] as shown in Figure 1B. However, thermal emission is a broadband phenomenon covering the infrared wavelength range of 5–30 μm (at 300 K temperature) [1], [2]. To enable the bandwidth to the entire thermal emission wavelength range (5–30 μm), two challenges arise. First, from the material perspective, the available constituent materials with ENZ wavelength (the wavelength where the real part of permittivity is zero) covering the entire thermal emission wavelength range are limited. Second, from the design perspective, to expand bandwidth, the number of ENZ thin-film layers needs to be drastically increased. However, undesired omnidirectional modes in multilayer stacking will be excited with increased thickness [31], [35]. Therefore, the aforementioned materials cannot be stacked or doped indefinitely. The above two challenges impose a fundamental limitation for the realization of BDTE with full coverage of the thermal band (e.g., 5–30 μm). Consequently, there is a lack of a universal approach to achieve directional thermal emission over the entire thermal emission wavelength range.

Here, we develop a general approach to achieve BDTE based on effective medium theory. We establish a universal theoretical model of designing broadband directional thermal emitters and show that BDTE can be realized beyond the previously considered ENZ and Berreman mode region [[Bibr j_nanoph-2023-0742_ref_031] [Bibr j_nanoph-2023-0742_ref_031] [Bibr j_nanoph-2023-0742_ref_032] [Bibr j_nanoph-2023-0742_ref_033] [Bibr j_nanoph-2023-0742_ref_034]. By applying the theory to different combinations of materials and nanostructures, directional thermal emitters with an ultra-broadband (5–30 μm) or arbitrary discrete bands (such as the two atmospheric windows) can be realized with only two materials. Furthermore, we show various broadband directional thermal emitters that exhibit strong (emissivity >0.8) directional (75–85°) emission over an ultra-broad spectral range (5–30 μm). It allows us to capture and utilize light and heat energy comprehensively. It has potential applications for high-efficiency information encryption, energy collection and utilization, thermal camouflaging, and infrared detection.

## 2 Results and discussion

We start by considering a typical directional thermal emitter design as shown in Figure 1A, which consists of an absorbing medium of thickness *d* on a metal reflector. We choose some typical thicknesses of monolayer absorbing medium taken from previous research, such as *d* = 0.1, 0.4 μm [[Bibr j_nanoph-2023-0742_ref_031] [Bibr j_nanoph-2023-0742_ref_032] [Bibr j_nanoph-2023-0742_ref_033] [Bibr j_nanoph-2023-0742_ref_034] and a larger thickness of *d* = 2 μm. To explore the full parameter space without being constrained by materials, we allow the permittivity of the absorbing medium to be arbitrarily varying. We define a figure of merit (FOM) for the directional emission for directionality [40]:

(1) FOM(λ)=ΔE/Δθ

where *λ* is the incident wavelength, *E* is the emissivity of the system, Δ*E* = *E*_max_ − *E*_min_ denotes the contrast of the emissivity with different *θ*. Δ*θ* is defined as the full-width half maximum (*FWHM*) of *E*(*θ*), which denotes the angular distribution of the main emissivity peak at a specific *λ*. For each combination of wavelengths and permittivity, the corresponding FOM can be calculated according to Eq. (1) with a given *d*. The magnitude of FOM indicates the strength of the directionality of the thermal emission (a high FOM indicates a better directionality). To evaluate the broadband directional performance, we calculate the FOM for each combination of wavelengths and permittivity covering the entire thermal emission wavelength range. Here, we specify a range of desired FOM (the range in which directionality meets the requirements): 1 to 12, FOM = 1/rad: Δ*E* = 0.6 and Δ*θ* = 30° and FOM = 12/rad: Δ*E* = 1 and Δ*θ* = 5° (approximate results for simplicity). We show the permittivity of the top absorbing medium that supports maximum emissivity greater than 0.8 and FOM between 1 and 12/rad as indicated by the clusters of colored points for the TM polarization when *d* = 0.1 (Figure 1C), 0.4 (Figure 1D), and 2 μm (Figure 1E). The colors of the scattering points represent the magnitude of the FOM, and the coordinates represent the corresponding wavelength and permittivity.

As shown in Figure 1C–E, the curves represent the wavelength-dependent permittivity of the typical ENZ materials (blue curves: SiO_2_ [31], [32], black curves: InAs [34]) used in the corresponding references [31], [34] as one layer of the absorbing medium that support Berreman mode and the metamaterials (red curves) with appropriate structure parameters designed in our work with *d* = 0.1 (Figure 1C), 0.4 (Figure 1D), and 2 μm (Figure 1E). Here, for ease of comparison, we only select the permittivity in the wavelength ranges that support a maximum emissivity greater than 0.8 (*E*_max_ > 0.8) for different thicknesses. The span of the curves on the *z*-axis is the wavelength range supporting *E*_max_ > 0.8. The portions of these curves that overlap with the FOM point clusters give the FOM magnitude of the thermal emission supported by the emitter when this material is used as an absorbing medium. The portions of the curves outside the clusters of colored points indicate the permittivity supporting either undesired FOM or low *E*_max_.

For comparison, the permittivity of the top absorbing medium that supports the aforementioned desired emission (indicated by the colored point clusters) shows a much broader range than the permittivity range of ENZ Berreman mode considered in previous research. Meanwhile, directional thermal emitters based on a monolayer of the commonly utilized ENZ material exhibit satisfactory performance only in restricted wavelength ranges. To enable strong directional thermal emission over an ultra-broad wavelength range, it is desired to regulate the permittivity throughout the intended wavelength range. Here, we utilize multiphase composite metamaterials with multiple structures based on effective medium theory (EMT) to flatten the permittivity of the absorbing medium in the high FOM area over the entire thermal emission wavelength range. For different materials, there is a range of thicknesses that are suitable in an actual system (Figure 1A) to achieve broadband directional thermal emission. Based on the approach proposed in this paper, for different thicknesses that lie within this acceptable range, as shown in Figure 1C–E, different metamaterials (as absorbing medium), with their permittivity flattened in the high FOM region over a broad wavelength range, can be designed to realize high-quality ultra-broadband directional thermal emission. The permittivity demonstrated here can support ultra-broadband directional thermal emission over 5–30 μm (or the wavelength range even broader). This will be further discussed in detail in Section 2.2.

### 2.1 General design principle of two-phase broadband directional thermal emitters

**Figure 2:**
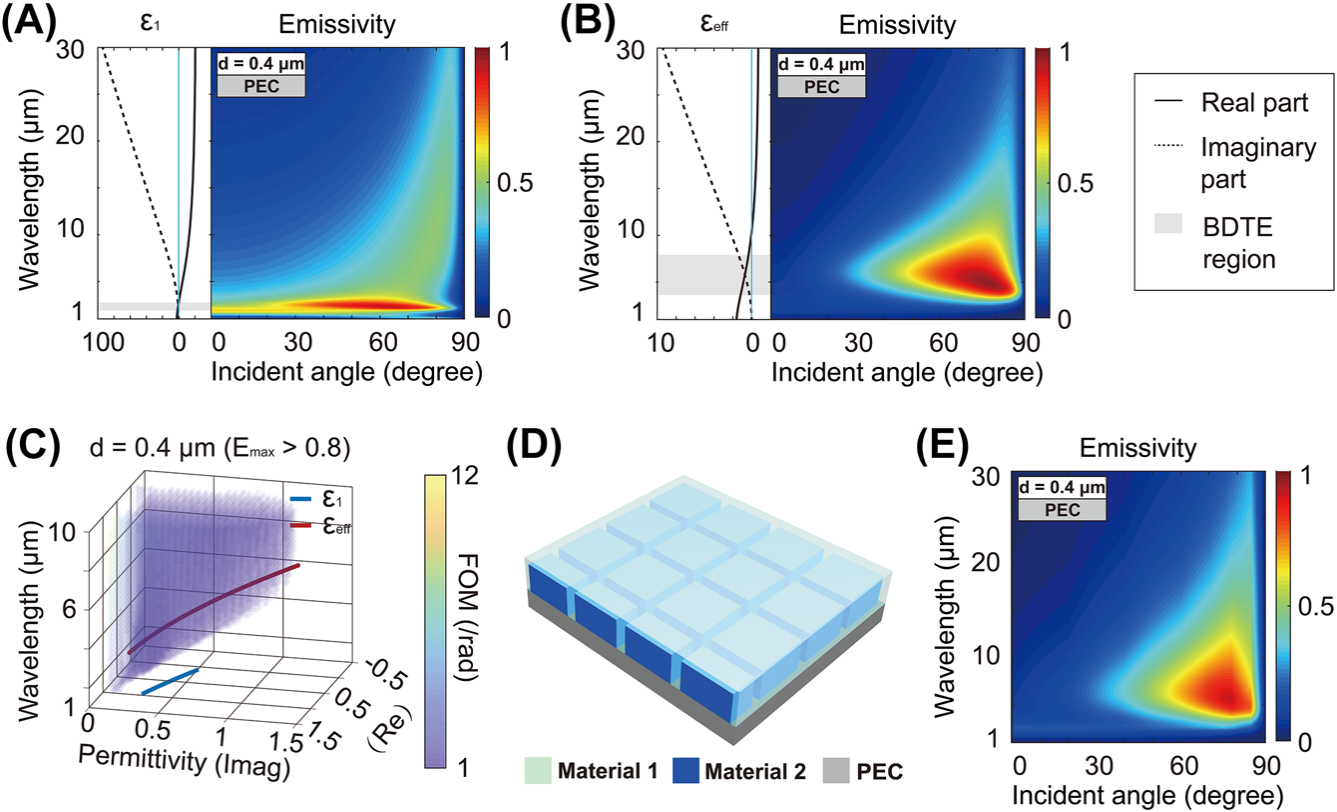
General design principle of two-phase ENZ metamaterials for broadband directional thermal emission. (A) The permittivity of the pure material described by the Drude model and the corresponding emissivity of the emitter based on this pure material calculated by TMM for the TM polarization. Here, *ɛ*_∞_ = 3, *λ*_*p*_ = 1.5 μm, *γ* = 0.2 *ω*_*p*_, *d* = 0.4 μm. (B) The effective permittivity of the two-phase ENZ metamaterial calculated by EMT and the corresponding emissivity of the emitter based on this two-phase metamaterial calculated by TMM. Here, *ɛ*_∞_ = 3, *λ*_*p*_ = 1.5 μm, *γ* = 0.2 *ω*_*p*_, *ɛ*_2_ = 1.5, *d* = 0.4 μm, *f* = 0.9, *f* corresponds to the volume fraction of material 2. (C) When the thickness of the top absorbing medium *d* = 0.4 μm, the clusters of colored points indicate the permittivity of the top absorbing medium supporting maximum emissivity above 0.7 and FOM between 1 and 12/rad. The curves represent the wavelength-dependent permittivity of the materials (blue curve: pure material 1, red curve: two-phase composite consisting of material 1 and 2 with *f* = 0.9) as the absorbing medium. (D) Schematic representations of a corresponding realistic structure. The green, blue, and dark gray areas represent materials 1, 2, and PEC, respectively. (E) Full-wave simulation of emissivity of the emitter that is shown in (D), showing a good agreement with EMT (B). The structural parameters of the emitter are detailed in the Supplementary Note 8.

To precisely regulate the permittivity of the absorbing medium, without losing generality, we begin with common conducting materials with their relative permittivity (*ɛ*_1_) described by the Drude model as Eq. (2):

(2) $$\varepsilon_{1}=\varepsilon_{\infty }-\frac{\omega_{p}^{2}}{\omega \left(\omega +i\gamma \right)}=\varepsilon_{1}^{\prime }{+\varepsilon }_{1}^{\prime \prime }i$$

where *ɛ*_∞_ is the high-frequency dielectric constant, *ω* is the operating angle frequency, *γ* is the collision frequency, and *ω*_*p*_ is the plasma frequency. For example, we take the plasma wavelength *λ*_*p*_ = 2*πc*/*ω*_*p*_ = 1.5 μm (*c* is the speed of light), *ɛ*_∞_ = 3 and *γ* = 0.2 *ω*_*p*_, *d* = 0.4 μm. The permittivity of this pure material (*ɛ*_1_) described by the Drude model and the emissivity calculated by the transfer matrix method (TMM) [31], [32] are shown in Figure 2A. For pure conducting materials, there is a narrow ENZ region and consequently a narrow region of directional thermal emission in the short-wavelength range due to the large slope of its permittivity near the ENZ wavelength. Meanwhile, the permittivity of such materials is generally negative in the long-wavelength region.

To red shift the ENZ region, we dilute this material with a dielectric (material 2) having a positive permittivity *ɛ*_2_, forming a metamaterial [41–43]. In this scenario, the effective medium theory is considered to extract the effective permittivity of the metamaterial. We take the plasma wavelength *λ*_*p*_ = 1.5 μm, *ɛ*_∞_ = 3, *γ* = 0.2 *ω*_*p*_ for material 1; *ɛ*_2_ = 1.5 for material 2 [44], [45], [46], the volume fraction of material 2 (*f*) as 0.9, *d* = 0.4 μm. According to the Maxwell-Garnett effective medium theory, the effective permittivity of the two-phase composite (*ɛ*_eff_) calculated by EMT [47], [48] and the emissivity calculated by TMM is shown in Figure 2B. These results indicate that the high emissivity (>0.8) can be obtained from 60° to 80° directionally and 4 μm–8 μm spectrally, much broader than that in the case of Figure 2A. Figure 2C illustrates the permittivity range at different wavelengths for emitters of *d* = 0.4 μm that satisfies the maximum emissivity greater than 0.7 and FOM between 2 and 12/rad. Here, the two curves represent the permittivity of the pure material 1 and the two-phase mixture described above for wavelengths with emissivity greater than 0.7. After the dilution, the ENZ wavelength of the composite moves to a longer wavelength than that of the pure material 1.

For common conducting material, the slope of permittivity in the ENZ region is large. Therefore, the ENZ region is narrow, which leads to a narrow wavelength range of high emissivity. However, for the two-phase metamaterial after the dilution aforementioned, the reduced slope of effective permittivity effectively expands the ENZ region, which allows a larger bandwidth of directional thermal emission. In general, the greater the volume fraction of the dielectric, the longer the ENZ wavelength, the smaller the slope of the effective permittivity, the broader the ENZ region, and the larger the bandwidth of the directional thermal emission. Evidently, by adjusting *f*, high emissivity at the target spectral regions can be obtained. Conversely, the *f* required can be derived if we aim for the real part of the effective permittivity to be zero at a particular wavelength as evidenced in Supplementary Note 1. The effective medium approximation presented depicts the envisioned structure of the actual system as illustrated in Figure 2D. The realistic structure of the absorbing medium can be understood by considering material 1 containing the subwavelength particles of material 2. We set the bottom metal layer as perfect electrical conductor (PEC), the thickness of the two-phase metamaterial *d* = 0.4 μm, the detailed structural parameters of the unit cell can be found in Supplementary Note 8. The full-wave simulation of emissivity based on the frequency domain finite element method (CST STUDIO SUITE) for the TM polarization of this system, as shown in Figure 2E, agrees well with EMT (Figure 2B), proving the validity of our theoretical model.

Additionally, we attempt to implement spatially gradient ENZ materials to realize BDTE. As schematically displayed in Supplementary Note 2, the broadband directional thermal emitters are constructed by stacking the layers of two-phase ENZ composites with different degrees of dilution (*f*_*n*_), the volume fraction of material 2 of each layer that varies spatially along the depth and thickness (*h*_*n*_, the thickness of each layer). Based on the two-phase composites consisting of the stacked layers of theoretical materials, the directional thermal emitters with a broadband (5–20 μm) or discrete bands (such as the two atmospheric windows) are conceivable. However, such stacking of effective medium in realistic structures is challenging. Moreover, for longer wavelengths, the directional thermal emitters based on two-phase composites require extremely large *f*. For example, the required *f* is larger than 0.99 if we aim for the ENZ wavelength of the two-phase composite up to 30 μm for the case shown in Figure 2A. This presents a challenge for fabrication and the expansion of the high emissivity waveband.

### 2.2 Ultra-broadband directional thermal emitter based on three-phase metamaterials

**Figure 3:**
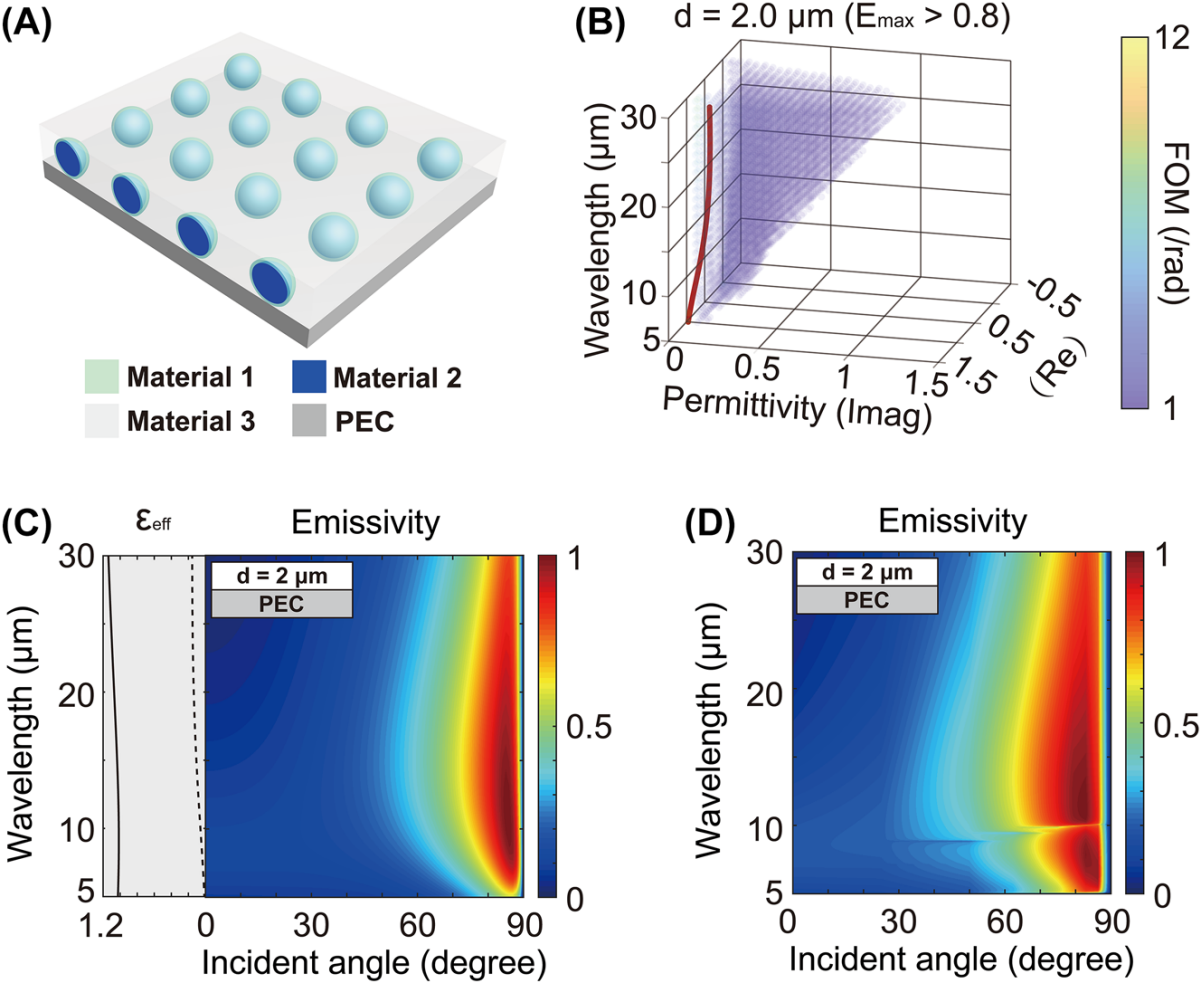
Ultra-broadband directional thermal emitter based on three-phase metamaterials. (A) Schematic of an emitter consisting of a PEC layer and a top absorbing medium made of a three-phase metamaterial. The three-phase metamaterial is made of a core–shell structure array (material 1: shell; material 2: core) mixed with material 3. (B) When the thickness of the top absorbing medium *d* = 2 μm, the clusters of colored points indicate the permittivity of a top absorbing medium that supports maximum emissivity greater than 0.8 and FOM between 2 and 12/rad for the TM polarization. The curves represent the effective permittivity of the three-phase composite (A) consisting of material 1 (*ɛ*_∞_ = 3, *λ*_p_ = 1.5 μm, *γ* = 0.2 *ω*_p_), material 2 (*ɛ*_2_ = 1.5), and material 3 (*ɛ*_2_ = 1) as the absorbing medium. The core diameter is 1 μm, the shell thickness is 0.17 μm, and the periodicity is 5 μm. The permittivity of the absorbing medium is flattened in the high FOM region over 5–30 μm. (C) The effective permittivity of the absorbing medium and the corresponding emissivity of the emitters based on three-phase composites calculated by EMT and TMM (solid black line: real part of the effective permittivity, dashed line: imaginary part, gray block: BDTE region). The BDTE region covers the entire thermal band. (D) Full-wave simulation of emissivity, showing a good agreement with EMT.

To further expand the BDTE bandwidth, we integrate an additional dielectric (material 3, *ɛ*_3_) into the design of three-phase composite metamaterials. We design the absorbing medium as shown in Figure 3A. The three-phase metamaterial is made of a core–shell structure array (material 1: shell; material 2: core) mixed with material 3 [49], [50]. Here, *λ*_*p*_ = 1.5 μm, *ɛ*_∞_ = 3, *γ* = 0.2 *ω*_*p*_ for material 1; *ɛ*_2_ = 1.5 for material 2 [44], [45], [46]; *ɛ*_3_ = 1 for material 3, *d* = 2 μm. The core diameter is 1 μm, the shell thickness is 0.17 μm, and the periodicity is 5 μm. The effective permittivity of the three-phase metamaterial is shown in Figure 3B and C calculated by the methods in Ref. [49]. When the thickness of the top absorbing medium *d* = 2 μm, the clusters of colored points indicate the permittivity of the top absorbing medium that supports maximum emissivity greater than 0.8 and FOM between 2 and 12/rad for the TM polarization. The curves represent the effective permittivity of the absorbing medium made of the three-phase metamaterial consisting of the aforementioned materials 1–3 (as illustrated in Figure 3A). The real part remains slightly greater than 1, while the imaginary part remains small. The slope of the effective permittivity becomes remarkably small, which allows us to smooth the permittivity of the absorbing medium in the high FOM region over an ultra-broad wavelength range. The high emissivity ( > 0.8) exists from 5 μm to 30 μm spectrally and 75°–88° directionally. The calculated emissivity for the TM polarization based on EMT and TMM (Figure 3C) agrees well with the calculations of the full-wave simulation as in Figure 3D.

The high emissivity regions of previous broadband directional thermal emitters based on the Berreman mode occur at large angles [[Bibr j_nanoph-2023-0742_ref_031] [Bibr j_nanoph-2023-0742_ref_032] [Bibr j_nanoph-2023-0742_ref_033] [Bibr j_nanoph-2023-0742_ref_034] due to the phase matching condition [39]. From another perspective, according to coupled-mode theory, the nonradiative loss of the three-phase metamaterial proposed in our paper is relatively small. Therefore, the system can reach the state of critical coupling in the large angle range (the radiation loss is small), which can lead to high emissivity [51]. For TE and TM polarization, the localizations of the system for the electric field are significantly different. A discussion of the polarization selectivity of the emitters can be found in Supplementary Note 7.

The emissivity spectra can be tuned by varying the intrinsic properties and volume fractions of the materials. On the one hand, an ultra-broadband high emissivity can be obtained by tailoring the volume fraction of dielectrics. On the other hand, with the same thickness, the thermal emission with directions skewing to larger angles can be obtained due to the smaller slope of the effective permittivity, especially in the case of three-phase composites (Supplementary Note 3–4). These characteristics can reduce the influence of additional undesired omnidirectional modes in multilayer stacking with increased thickness and thus enable the maximal directional contrast and the ultra-broad spectral regions of the high emissivity for the TM-polarized light at the expense of the tunability to near-normal angles of incidence. For a detailed explanation of this section, please refer to Supplementary Note 3–4.

### 2.3 Structures of alternative three-phase ultra-broadband directional thermal emitters

**Figure 4:**
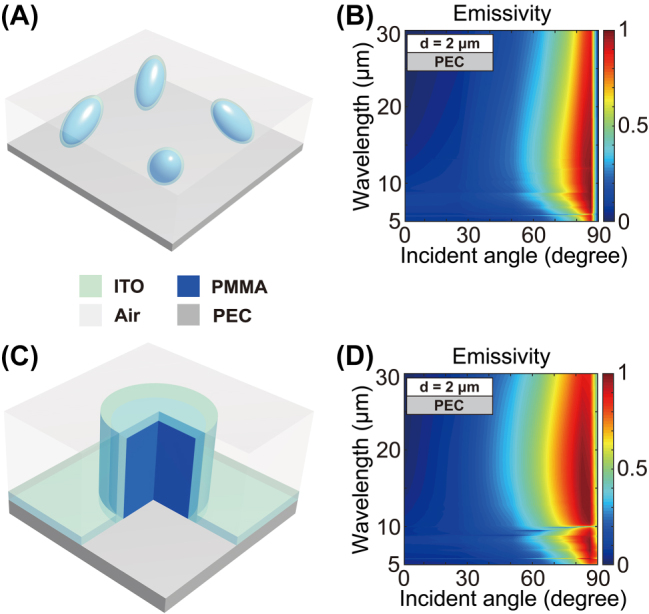
Alternative structures of three-phase metamaterials supporting ultra-broadband directional thermal emission. (A and C) Schematic of an emitter consisting of a PEC layer and a top absorbing medium made of a three-phase metamaterial. (A) The three-phase metamaterial is made of an ellipsoid core–shell structure array (ITO: shell; PMMA: core) mixed with air. The major axes of the ellipsoid core–shells are randomly oriented. (C) The emitter with absorbing medium consisting of a cylinder array (PMMA) covered by a conformal coating of ITO, mixed with air. (B and D) Full-wave simulations of emissivity for emitters as shown in (A–C) for the TM polarization. Alternative structures designed based on our method are capable of achieving strong directional thermal emission in the ultra-broad wavelength range. Detailed structural parameters can be found in the Supplementary Note 8.

Based on the above-discussed thermal emitters with concentric spheres as periodic structural units, we further show multiple alternative emitter designs that all exhibit ultra-broadband directional thermal emission features [52], [53]. For actual manufacturing, we present these designs based on realistic materials. We consider indium tin oxide (ITO) as material 1. The ENZ wavelength of ITO occurs at near-infrared wavelengths and can be tuned by controlling the doping density and applying a static electric field [46, 54, 55]. The permittivity of ITO (*ɛ*_*ITO*_) is well described by the Drude model as *λ*_*p*_ = 1.12 *μm*, *ɛ*_∞_ = 3.9, *γ* = 0.1256 *ω*_*p*_ [55]. We use PMMA [56] as material 2 and air (*ɛ*_*air*_ = 1) as material 3 for simplicity. The material of the bottom mirror is silver whose permittivity (*ɛ*_*silver*_) is described by the Drude model as *λ*_*p*_ = 0.139 µm, *ɛ*_∞_ = 5, *γ* = 0.0041 *ω*_*p*_ [57]. The first example includes an absorbing medium formed by an ellipsoid core–shell structure array (ITO: shell; PMMA: core) mixed with air. The major axes of the ellipsoid core–shells are randomly oriented. The unit cell is shown in Figure 4A. We perform full-wave simulations of emissivity for five cases: we arbitrarily change the orientation of the long axis of the ellipsoid core–shells for each full-wave simulation. For each different incident wavelength and angle, we average the emissivity of the five simulations, and the average results obtained are shown in Figure 4B. The full-wave simulation results show that the high emissivity (>0.8) exists from 5 μm to 30 μm spectrally and 80°–87° directionally.

We further consider a second example that is more experimentally feasible. The absorbing medium is formed by a periodic array of subwavelength dielectric cylinders (PMMA) covered by a conformal coating of ITO layer. The rest is filled by air. Figure 4C is a schematic illustration of the unit cell. Detailed structural parameters can be found in Supplementary Note 8. The full-wave simulation results show that the high emissivity ( > 0.8) exists from 5 μm to 30 μm spectrally and 76°–89° directionally.

Considering the unavoidable imperfections in the process of manufacturing, we further verify the robustness of this approach by calculating the emissivity of emitters with structural perturbations and different shapes of dielectrics. It is confirmed that comparable outcomes can be achieved for the unit cell with equal volume fractions and thicknesses. See Supplementary Note 6 for a detailed explanation of this section. Moreover, based on this approach, we can choose various materials and different volume fractions (i.e., the structural parameters) to achieve the desired BDTE. In addition, all the above-mentioned emitters present very low emissivity in the transverse electric (TE) polarization, indicating excellent polarization selectivity BDTE (see Supplementary Note 6).

## 3 Discussions

We establish a universal approach based on effective medium theory to realizing ultra-broadband directional thermal emitter without being constrained by materials. We show BDTE can be realized beyond the previously considered epsilon-near-zero and Berreman mode region. We numerically demonstrate strong (>0.8) directional (80 ± 5°) thermal emission covering the entire thermal emission wavelength range (5–30 μm) by using only two materials. The appropriate materials and volume fractions can be chosen to achieve the BDTE covering various wavebands of high emissivity. For the emitters based on the absorbing medium/metal reflector system as shown in Figure 1A, the angular range of the high emissivity region can be controlled by appropriately increasing the thickness of the absorbing medium [31], [32], decreasing the intrinsic loss of the absorbing medium [31], [58], adding dielectric spacers [58], using magneto-optical materials [34], [58], and designing surface structures [27], [59]. Meanwhile, the bandwidth of directional thermal emission can be broadened through the methods of gradient ENZ and effective medium theory. Our results indicate new opportunities for designing thermal emitters for potential applications such as information encryption, energy collection and utilization, thermal camouflaging (providing additional cooling capability), infrared detection (determining or limiting the range of positions of target objects), sensing, and imaging. Moreover, the concept of achieving ENZ behavior in a suitable waveband introduces novel opportunities to investigate the extraordinary physical phenomena of ENZ materials, such as electromagnetic tunneling [60], nonlinear enhancement [61], [62], optical nonreciprocity [63], and Casimir interaction [64].

## 4 Methods

### 4.1 Metamaterial design and theoretical model

First, we determine the wavelength ranges of the desired directional emission. Based on the transmission matrix method (the substrate: PEC), the wavelength, the real and imaginary parts of the permittivity for the absorbing medium are scanned to calculate the corresponding FOM. On this basis, the desired range of effective permittivity can be determined. Then we choose the appropriate materials and volume fractions, i.e., the Drude model parameters, permittivity of the dielectric, volume fractions (for two-phase metamaterials), and structural parameters (for three-phase metamaterials). For metamaterials with different structures, the effective permittivity can be calculated by the corresponding effective medium approximation. The reflectivity of the system is calculated by substituting this effective permittivity into the transmission matrix. According to Kirchhoff’s law, the emissivity of a system is exactly equal to its absorptivity within thermal equilibrium. The absorptivity is equivalent to 1 minus the reflectivity (*R*) and transmissivity (*T*). *T* is equal to zero due to the presence of a reflector at the bottom of the emitter. The emissivity of system *E* = 1 − *R*.

All numerical simulations in this study were performed using the commercial software package (CST STUDIO SUITE, Frequency Domain Solver). To verify the correctness of the theoretical model based on the effective medium approximation, optical simulations of three-dimensional models of the unit cells were implemented. For CST STUDIO SUITE, the boundary conditions settings: unit cell condition in the *X* and *Y* direction, open boundary (add space) in the *Z*_max_ direction, and electric boundary (*E*_*t*_ = 0) in the *Z*_min_ direction. The optical response of metamaterial was analyzed by considering the light entering with a specific polarization and a series of wavelengths and incident angles. To ensure accurate calculations, a computational accuracy of 1E-6 was used to calculate the effect of incident angle and wavelength on emissivity.

## Supplementary Material

Supplementary Material Details
