# Supplementary material for: Ultra-broadband directional thermal emission
Source: Nanophotonics. 2024 Jan 16;13(5):793–801. doi: 10.1515/nanoph-2023-0742 (PMC11501114; doi:10.1515/nanoph-2023-0742)
Supplement: Supplementary file 1 — Supplementary Material Details [file j_nanoph-2023-0742_suppl_001.pdf]

Supporting Information

Ultra-broadband directional thermal emission

Qiuyu Wang [a, b], Tianji Liu [a, b], Longnan Li [a, b], Chen Huang [a, b], Jiawei Wang [a, b], Meng Xiao [c], Yang Li [d], Wei Li [a, b *]

[a] *GPL Photonics Laboratory, State Key Laboratory of Luminescence and Applications, Changchun Institute of Optics, Fine Mechanics and Physics, Chinese Academy of Sciences, Changchun 130033, China*

[b] *University of Chinese Academy of Sciences, Beijing 100039, China*

[c] *Key Laboratory of Artificial Micro- and Nano-Structures of Ministry of Education and School of Physics and Technology, Wuhan University, Wuhan 430072, China*

[d] *State Key Laboratory of Precision Measurement Technology and Instruments, Department of Precision Instrument, Tsinghua University, Beijing, 100084 China*

[*] Correspondence: weili1@ciomp.ac.cn

Supplementary Note 1: Calculation of the desired volume fraction for two-phase metamaterial.

According to the Maxwell-Garnett effective medium theory, the effective permittivity (ε_{pd}) of the two-phase metamaterial can be written as:

$$\frac{\varepsilon_{pd}-\varepsilon_1}{\varepsilon_{pd}+2\varepsilon_1} = f\frac{\varepsilon_2-\varepsilon_1}{\varepsilon_2+2\varepsilon_1} \tag{S1}$$

$$\varepsilon_1 = \varepsilon_\infty - \frac{\omega_p^2}{\omega(\omega+i\gamma)} = \varepsilon_1' + \varepsilon_1'' i \qquad \text{(Eq. (1) in the main text)}$$

Here, we set ε_2 as a fixed positive real number. Note here ε_1 is a wavelength-dependent complex function. In this case, when the real part of the dielectric constant of the composite material is 0, the desired f_{ENZ} can be derived as:

$$f_{ENZ} = -[3({\varepsilon_2}^4{\varepsilon_1'}^2 + 2{\varepsilon_2}^3{\varepsilon_1''}^2\varepsilon_1' + 2{\varepsilon_2}^3{\varepsilon_1'}^3 + 9{\varepsilon_2}^2{\varepsilon_1''}^4 + 6{\varepsilon_2}^2{\varepsilon_1''}^2{\varepsilon_1'}^2 - 3{\varepsilon_2}^2{\varepsilon_1'}^4 - 4\varepsilon_2{\varepsilon_1''}^4\varepsilon_1' - 8\varepsilon_2{\varepsilon_1''}^2{\varepsilon_1'}^3 - 4\varepsilon_2{\varepsilon_1'}^5 + 4{\varepsilon_1''}^4{\varepsilon_1'}^2 + 8{\varepsilon_1''}^2{\varepsilon_1'}^4 + 4{\varepsilon_1'}^6)^{\frac{1}{2}} - 9\varepsilon_2{\varepsilon_1''}^2 - \varepsilon_2{\varepsilon_1'}^2 - {\varepsilon_2}^2\varepsilon_1' + 2{\varepsilon_1''}^2\varepsilon_1' + 2{\varepsilon_1'}^3]/[4({\varepsilon_1''}^2\varepsilon_1' - 2\varepsilon_2{\varepsilon_1'}^2 + {\varepsilon_1''}^2\varepsilon_1' + {\varepsilon_1'}^3)] \tag{S2}$$

That is, at the target wavelength (frequency), the real part of ε_{pd} can become zero by adjusting f and thus achieve epsilon-near-zero (ENZ) property in the specified band.

For example, when the ENZ wavelength is set as 10 μm, for $\varepsilon_\infty = 3,\ \lambda_p = 1.5\ \mu m,\ \gamma = 0.1\ \omega_p$, $\varepsilon_2 = 1.5$, ε_1 is equal to $-27.7692 + 20.5128i$ at 10 μm, the calculated f is 0.9232.

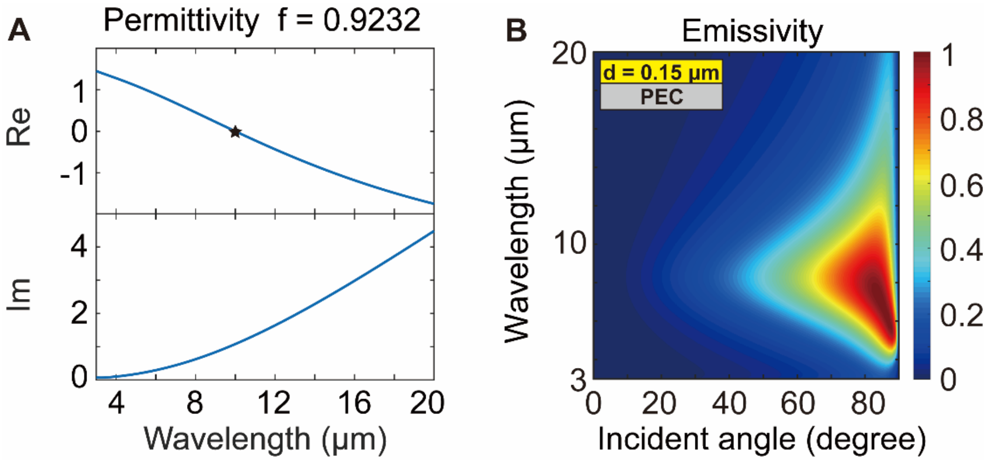

Figure S1: Calculation of the influence of the volume fraction. (A) Effective permittivity of the two-phase composite consists of material 1 ($\varepsilon_\infty = 3,\ \lambda_p = 1.5\ \mu m,\ \gamma = 0.1\ \omega_p$) and material 2 ($\varepsilon_2 = 1.5$), $f = 0.9232$ calculated by Eq. S2. The real part of the effective permittivity equals to zero at $10\ \mu m$. **(B)** Emissivity of the emitter based on this two-phase metamaterial with $d = 0.15\ \mu m$ for the TM polarization calculated by EMT and TMM. Directional thermal emission can be achieved in the ENZ region.

Supplementary Note 2: Two-phase absorbing medium based directional thermal emitters.

Figure S2A shows the real part and imaginary part of the effective permittivity of the two-phase composites (ε_{pd}) with different f calculated by the Drude model and EMT. Here, we take $\lambda_p = 1.5\,\mu m$, $\varepsilon_\infty = 3$, $\gamma = 0.1\,\omega_p$ for material 1 and $\varepsilon_2 = 1.5$ for material 2. For $f = 0.8$ and 0.95, the ENZ wavelength can be shifted from $2.7\,\mu m$ to $5.8\,\mu m$ and $13\,\mu m$. The emissivity of the emitters based on the composites above calculated by the EMT and TMM is shown in Figure S2B-C. The bandwidth of the spectral regions of high emissivity (> 0.8), is enlarged to $3\,\mu m$ and $9\,\mu m$. Evidently, for the TM polarization, the high emissivity at the target spectral regions can be obtained by adjusting f as Eq. (S2) in Supplementary Note 1.

Furthermore, we try to implement spatially gradient ENZ and thus BDTE. The broadband directional thermal emitters schematically displayed in Figure S2D are constructed by stacking the layers of two-phase ENZ composites with different degree of dilution, where f_n ($f_n > \cdots > f_2 > f_1$) represents the volume fraction of material 2 of each layer that varies spatially along the depth, and h_n ($h_n > \cdots > h_2 > h_1$) represents the thickness of each layer. The ENZ wavelength for the composites is shorter as the depth increases. When the permittivity of one composite layer reaches near zero, the composite layer underneath has a metal-like negative permittivity. The two adjacent composite layers form a configuration like that in Figure 1A. In this process, each layer works in tandem so that we can obtain the desired broad ENZ regions and thus high emissivity. We demonstrate broadband and discrete-band directional thermal emitters as shown in Figure S2E-F based on the two-phase composites consisting of the stacked layers of theoretical materials with $\lambda_p = 1.5\,\mu m$, $\varepsilon_\infty = 3$, $\gamma = 0.1\,\omega_p$ and $0.05\,\omega_p$, $\varepsilon_2 = 1.5$ by choosing the proper volume fractions based on Eq. (S1), respectively. The detailed parameters of the emitters are given in Supplementary Note 8. For the composites with longer ENZ wavelength, the thickness required is larger to obtain high emissivity at a certain angle.

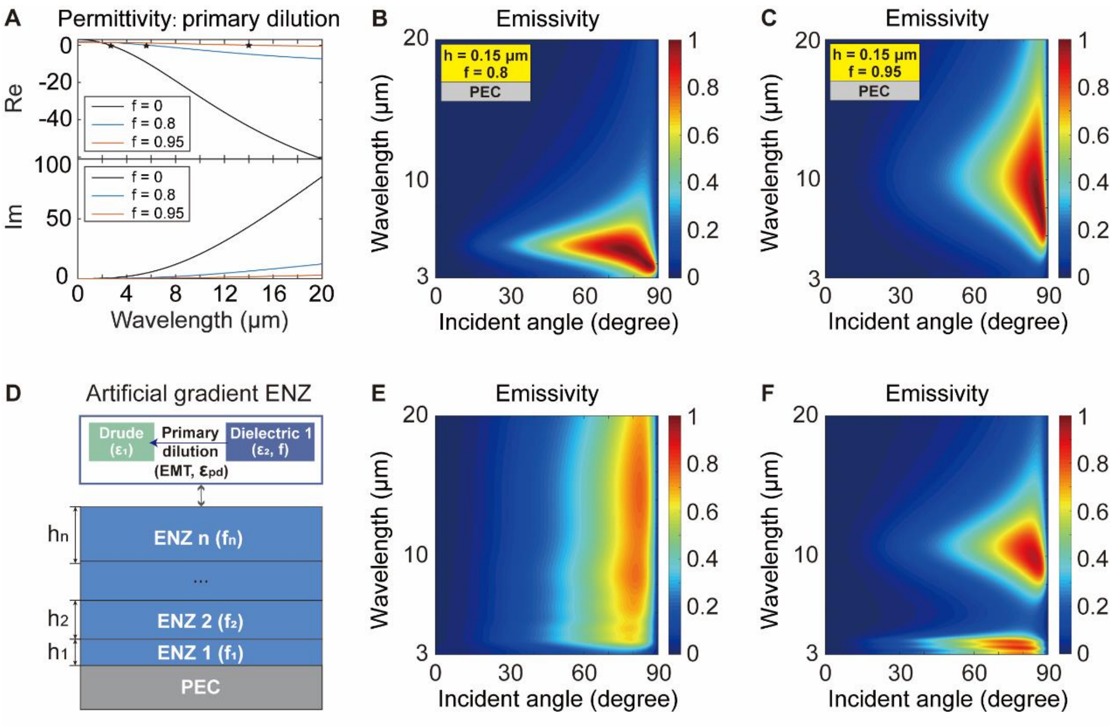

Figure S2: Thermal emission of the emitters based on ENZ two-phase composites for the TM polarization. (A) Effective permittivity of the two-phase composites consisting of material 1 ($\varepsilon_\infty = 3$, $\lambda_p = 1.5\ \mu m$, $\gamma = 0.1\ \omega_p$) and material 2 ($\varepsilon_2 = 1.5$) with different f calculated by EMT. **(B-C)** Emissivity of the emitters based on two-phase composites with $f = 0.8$ (B) and 0.95 (C) calculated by EMT and TMM. The thickness of the two-phase composites (d) is $0.15\ \mu m$. The spectrum response of the emitters can be designed by varying f. **(D)** Schematic of the emitters formed by stacking the two-phase composites consist of material 1 and 2 with different f on PEC substrate. **(E-F)** Calculated emissivity of the artificial gradient ENZ systems with different collision frequency used in material 1 , $\varepsilon_\infty = 3$, $\lambda_p = 1.5\ \mu m$, $\gamma = 0.1\ \omega_p$ **(E)** and $0.05\ \omega_p$ **(F)**, which can realize directional thermal emission over ultra-broad (3-20 μm) and discrete (3-5 and 8-13 μm) wavebands. The wavelength range of high emissivity can be designed flexibly by varying the degree of dilution.

Supplementary Note 3: Effect of material properties.

1. Material 1

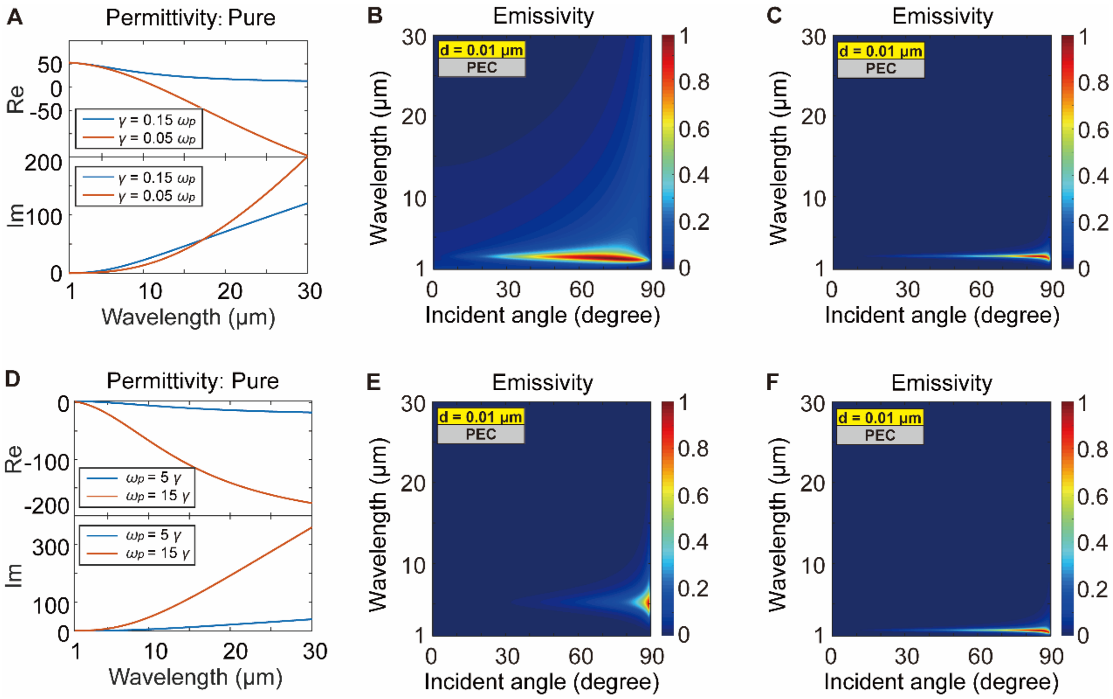

Figure S3: Effect of the plasma and collision frequency for pure material 1. (A) Permittivity of pure material 1 with $\varepsilon_\infty = 3$, $\lambda_p = 1.5\ \mu m$, $\omega_p = 1.2566 \times 10^{15}\ rad/s$, $\gamma = 0.15\ \omega_p$ and $0.05\ \omega_p$ calculated by Eq. 1. **(B-C)** Emissivity of the emitters based on these pure materials calculated by TMM for the TM polarization, $d = 0.01\ \mu m$. **(D)** Permittivity of pure material 1 with $\varepsilon_\infty = 3$, $\gamma = 1.2566 \times 10^{14}\ rad/s$, $\omega_p = 5\,\gamma$ and $15\,\gamma$ calculated by Eq. 1. **(E-F)** Emissivity of the emitter based on these pure materials calculated by TMM for the TM polarization, $d = 0.01\ \mu m$.

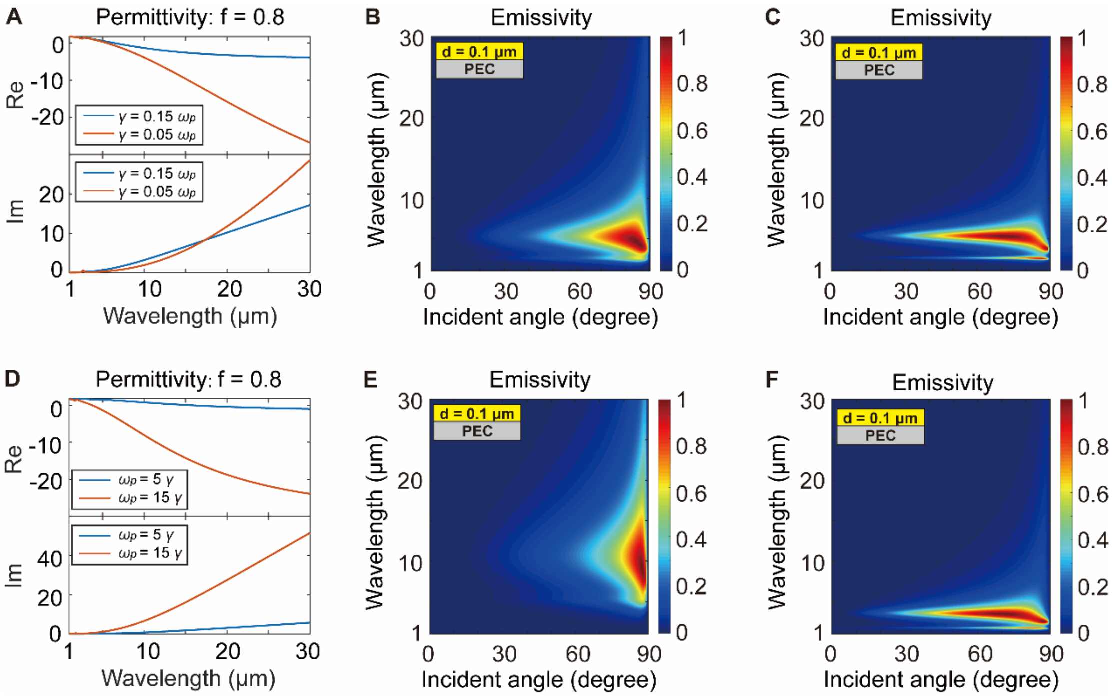

Figure S4: Effect of material 1 for two-phase metamaterials. (A) Effective permittivity of the two-phase metamaterials with $\varepsilon_2 = 1.5$, $f = 0.8$, $\varepsilon_\infty = 3$, $\lambda_p = 1.5\ \mu m$, $\omega_p = 1.2566 \times 10^{15}\ rad/s$, $\gamma = 0.15\ \omega_p$ or $0.05\ \omega_p$ calculated by Maxwell Garnett approximation [1] [2]. **(B-C)** Emissivity of the emitters based on these two-phase metamaterials calculated by TMM for the TM polarization, $d = 0.1\ \mu m$. **(D)** Effective permittivity of the two-phase metamaterials with $\varepsilon_2 = 1.5$, $f = 0.8$, $\varepsilon_\infty = 3$, $\gamma = 1.2566 \times 10^{14}\ rad/s$, $\omega_p = 5\ \gamma$ or $15\ \gamma$. **(E-F)** Emissivity of the emitter based on these two-phase metamaterials calculated by TMM for the TM polarization, $d = 0.1\ \mu m$.

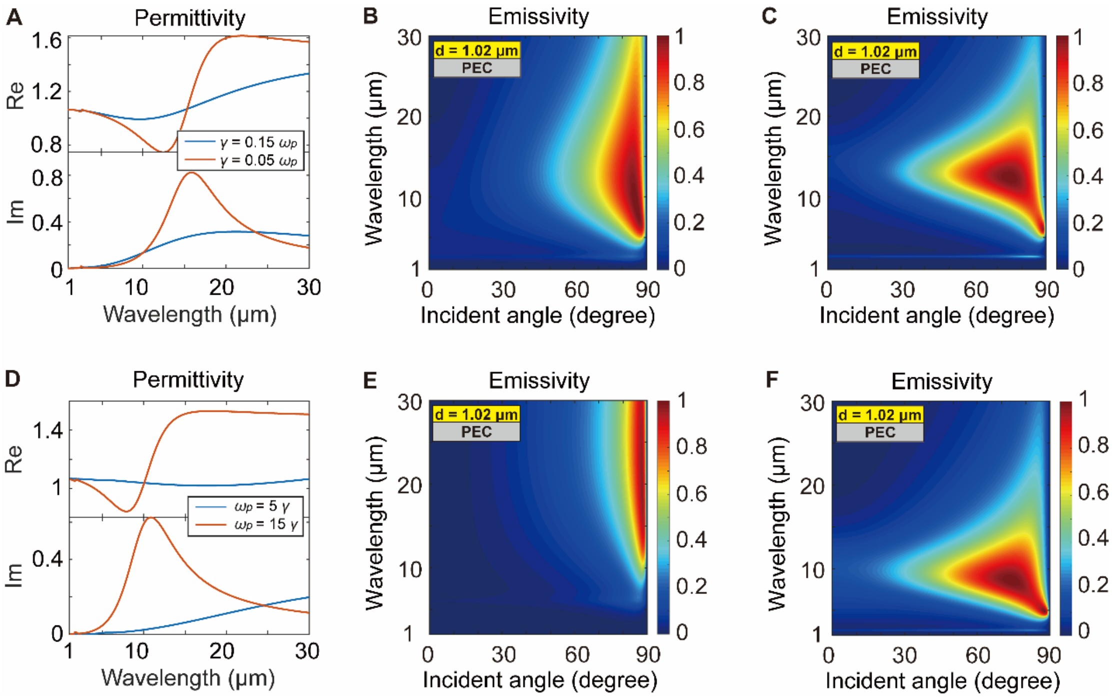

Figure S5: Effect of material 1 for three-phase metamaterials. The three-phase metamaterial is made of a core-shell structure array [3] (material 1: shell; material 2: core) mixed with material 3. The same structural parameters are used in this part. The core diameter is $0.5\ \mu m$, the shell thickness is $0.01\,\mu m$, and the periodicity is $2\,\mu m$. **(A)** Effective permittivity of the three-phase metamaterials with $\varepsilon_2 = 1.5$, $\varepsilon_3 = 1$, $\varepsilon_\infty = 3$, $\lambda_p = 1.5\,\mu m$, $\omega_p = 1.2566 \times 10^{15}\,rad/s$, $\gamma = 0.15\,\omega_p$ or $0.05\,\omega_p$ calculated by the methods in Ref. [3]. **(B-C)** Emissivity of the emitters based on these three-phase metamaterials calculated by TMM for the TM polarization, $d = 1.02\,\mu m$. **(D)** Effective permittivity of the three-phase metamaterials with $\varepsilon_2 = 1.5$, $\varepsilon_3 = 1$, $\varepsilon_\infty = 3$, $\gamma = 1.2566 \times 10^{14}\,rad/s$, $\omega_p = 5\,\gamma$ or $15\,\gamma$. **(E-F)** Emissivity of the emitter based on these two-phase metamaterials calculated by TMM for the TM polarization, $d = 1.02\,\mu m$.

2. Material 2

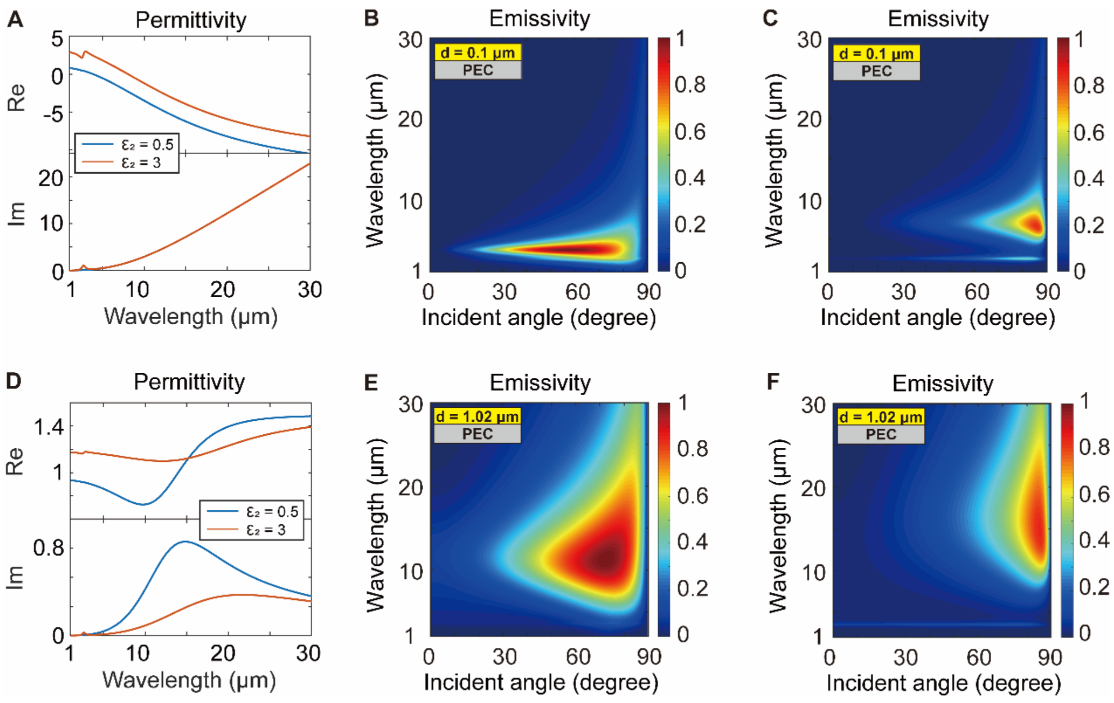

Figure S6. Effect of material 2 for the metamaterials. (A) Effective permittivity of the two-phase composites consisting of material 1 ($\varepsilon_\infty = 3$, $\lambda_p = 1.5\ \mu m$, $\gamma = 0.1\ \omega_p$) and 2 ($\varepsilon_2 = 0.5$ or 3), $f = 0.8$. **(B-C)** Emissivity of the emitters based on these two-phase composites calculated by EMT and TMM for the TM polarization, $d = 0.1\ \mu m$. **(D)** Effective permittivity of the three-phase composites consisting of material 1 ($\varepsilon_\infty = 3$, $\lambda_p = 1.5\ \mu m$, $\gamma = 0.1\ \omega_p$), 2 ($\varepsilon_2 = 0.5$ or 3), 3 ($\varepsilon_3 = 1$) calculated by the methods in Ref. [3]. The three-phase metamaterial is made of a core-shell structure array [3] (material 1: shell; material 2: core) mixed with material 3. The same structural parameters are used in this part. The core diameter is 0.5 μm, the shell thickness is $0.01\ \mu m$, and the periodicity is $2\ \mu m$. **(E-F)** Emissivity of the emitters based on these three-phase composites calculated by EMT and TMM for the TM polarization.

3. Material 3

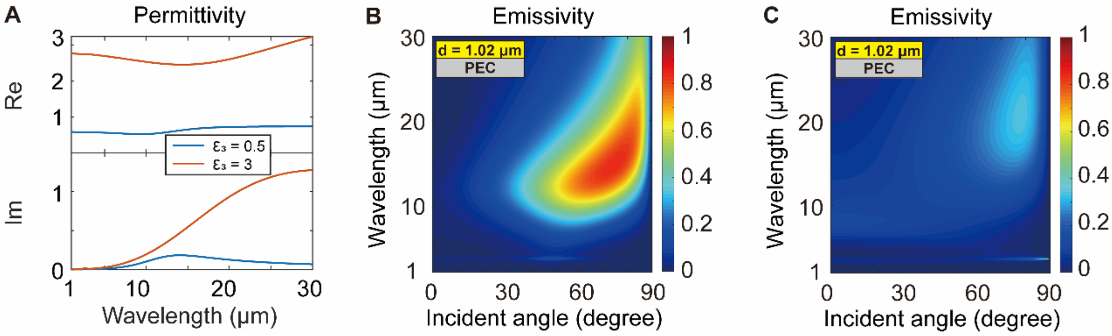

Figure S6. Effect of material 3 for three-phase metamaterials. The three-phase metamaterial is made of a core-shell structure array [3] (material 1: shell; material 2: core) mixed with material 3. The same structural parameters are used in this part. The core diameter is $0.5\ \mu m$, the shell thickness is $0.01\,\mu m$, and the periodicity is $2\,\mu m$. **(A)** Effective permittivity of the three-phase composites consisting of material 1 ($\varepsilon_\infty = 3$, $\lambda_p = 1.5\,\mu m$, $\gamma = 0.1\,\omega_p$), 2 ($\varepsilon_2 = 1.5$), 3 ($\varepsilon_3 = 0.5$ or 3) calculated by the methods in Ref. [3]. **(B-C)** Emissivity of the emitters based on these three-phase composites calculated by EMT and TMM for the TM polarization.

Supplementary Note 4: Effect of the structural parameters for three-phase absorbing medium.

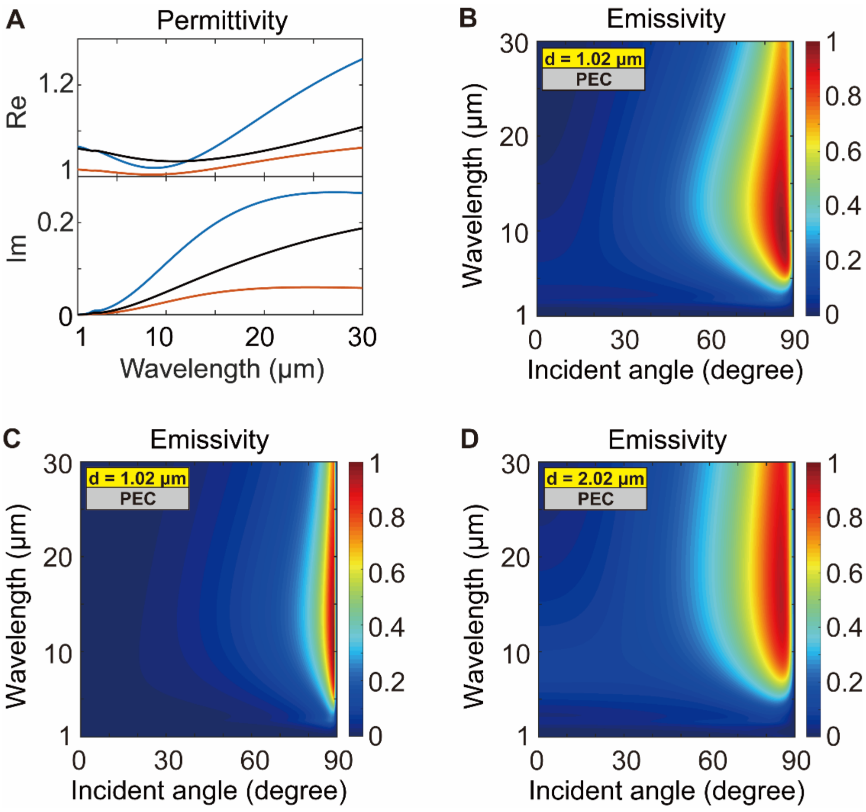

Figure S7. Effect of material 3 for three-phase metamaterials. The three-phase metamaterial is made of a core-shell structure array [3] (material 1: shell; material 2: core) mixed with material 3. **(A)** Effective permittivity of the three-phase composites consisting of material 1 ($\varepsilon_\infty = 3$, $\lambda_p = 1.5\ \mu m$, $\gamma = 0.1\ \omega_p$), 2 ($\varepsilon_2 = 1.5$), 3 ($\varepsilon_3 = 0.5$ or 3) calculated by the methods in Ref. [3]. Blue line: the core diameter is 0.5 μm, the shell thickness is $0.01\ \mu m$, and the periodicity is $2\ \mu m$. Red line: the core diameter is 0.5 μm, the shell thickness is $0.01\ \mu m$, and the periodicity is $4\ \mu m$ (the periodicity, i.e., the volume fraction of material 3 increases). Black line: the core diameter is 1 μm, the shell thickness is $0.01\ \mu m$, and the periodicity is $4\ \mu m$ (the core diameter increases, i.e., the volume fraction of material 3 decreases, the volume fraction of material 2 increases and the effective thickness d increases). **(B-D)** Emissivity of the emitters based on these three-phase composites above calculated by EMT and TMM for the TM polarization.

Supplementary Note 5: Verification of the causes of high emissivity.

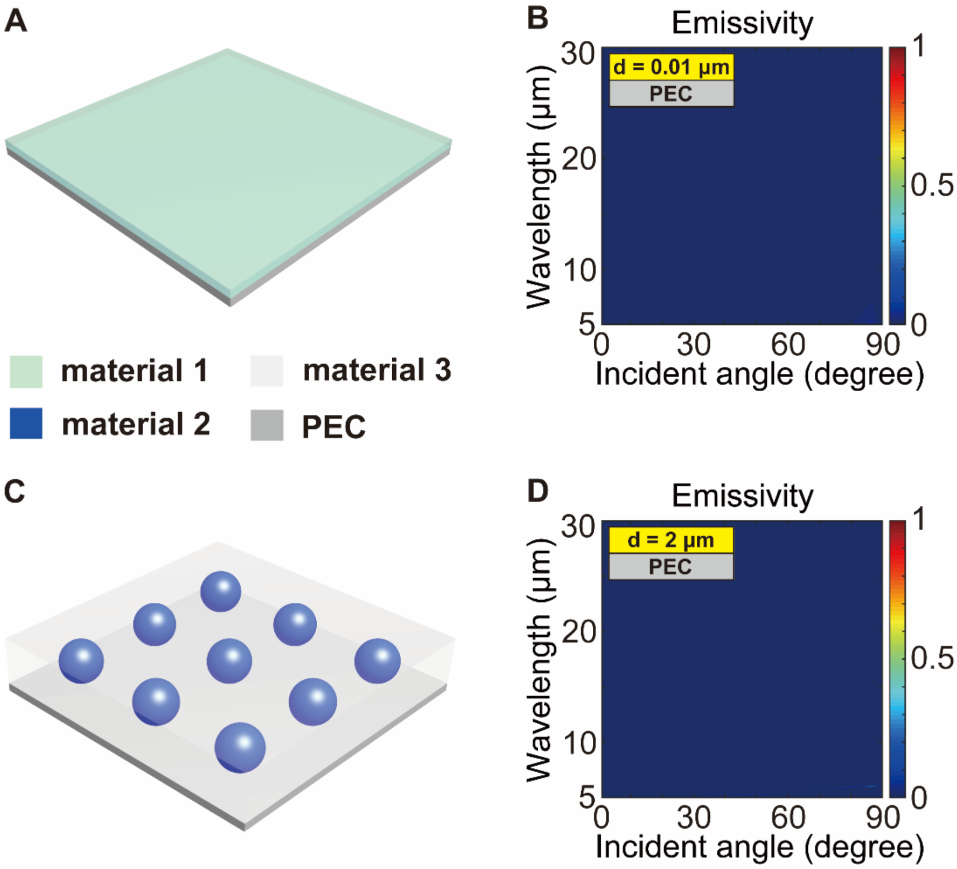

Figure S8: Verification of the causes of high emissivity. (A) Schematic of an emitter with the absorbing medium made of pure material 1. **(B)** Full-wave simulation of emissivity for the emitter based on pure material 1 ($\varepsilon_\infty = 3$, $\lambda_p = 1.5\ \mu m$, $\gamma = 0.1\ \omega_p$) for the TM polarization, $d = 0.01\ \mu m$. **(C)** Schematic of an emitter with the absorbing medium formed by a sphere array made of pure material 2 mixed in material 3. **(D)** Full-wave simulation of emissivity for the emitter ($\varepsilon_2 = 1.5$, $\varepsilon_3 = 1$) for the TM polarization, $d = 2\ \mu m$. The sphere diameter is 1 μm and the periodicity is $5\ \mu m$. The high emissivity within the ultra-broad waveband shown in Figure 3D comes from the combined effect of the two materials as well as the structure. The emissivity can be substantially increased according to our method.

Supplementary Note 6: Robustness against perturbations and geometric deformation.

1. Addition of perturbations

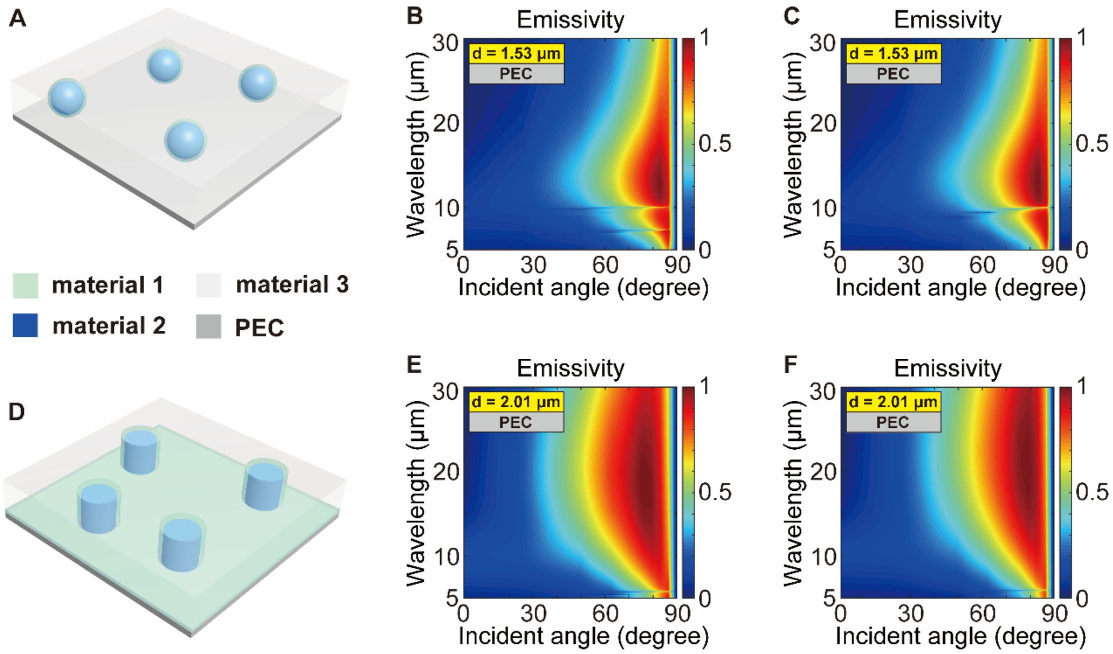

Figure S9: Verification of the causes of high emissivity. (A) Schematic of an emitter with the absorbing medium based on an array of randomly arranged spherical core shells (material 1: shell; material 2: core) mixed with material 3. The random arrangement of the spherical core-shell introduces perturbations into the system. **(B)** Full-wave simulation of emissivity for the corresponding emitter with aforementioned perturbations for the TM polarization. $\varepsilon_\infty = 3$, $\lambda_p = 1.5\ \mu m$, $\gamma = 0.1\ \omega_p$, $\varepsilon_2 = 1.5$, $\varepsilon_3 = 1$. The core diameter is $0.75\ \mu m$, the shell thickness is $0.015\ \mu m$, and the periodicity is $5\ \mu m$. **(C)** Full-wave simulation of emissivity for the corresponding emitter ($\varepsilon_\infty = 3$, $\lambda_p = 1.5\ \mu m$, $\gamma = 0.1\ \omega_p$, $\varepsilon_2 = 1.5$, $\varepsilon_3 = 1$) without aforementioned perturbations (spherical shells arranged periodically and regularly) for the TM polarization. The core diameter is $0.75\ \mu m$, the shell thickness is $0.015\ \mu m$, and the periodicity is $5\ \mu m$ (the same as **B**). **(D)** An emitter with absorbing medium consisting of randomly arranged multilayer cylinder structure array (material 1: layer; material 2: cylinder) mixed with material 3. **(E)** Full-wave simulation of emissivity for this emitter ($\varepsilon_\infty = 3$, $\lambda_p = 1.5\ \mu m$, $\gamma = 0.1\ \omega_p$, $\varepsilon_2 = 1.5$, $\varepsilon_3 = 1$) with aforementioned perturbations for the TM polarization, $d = 2.01\ \mu m$. **(F)** Full-wave simulation of emissivity for this emitter ($\varepsilon_\infty = 3$, $\lambda_p = 1.5\ \mu m$, $\gamma = 0.1\ \omega_p$, $\varepsilon_2 = 1.5$, $\varepsilon_3 = 1$) without aforementioned perturbations for the TM polarization (the same structural parameters as **(E)**). See Supplementary Note 8 for the detail parameters.

2. Unit cells with different shapes

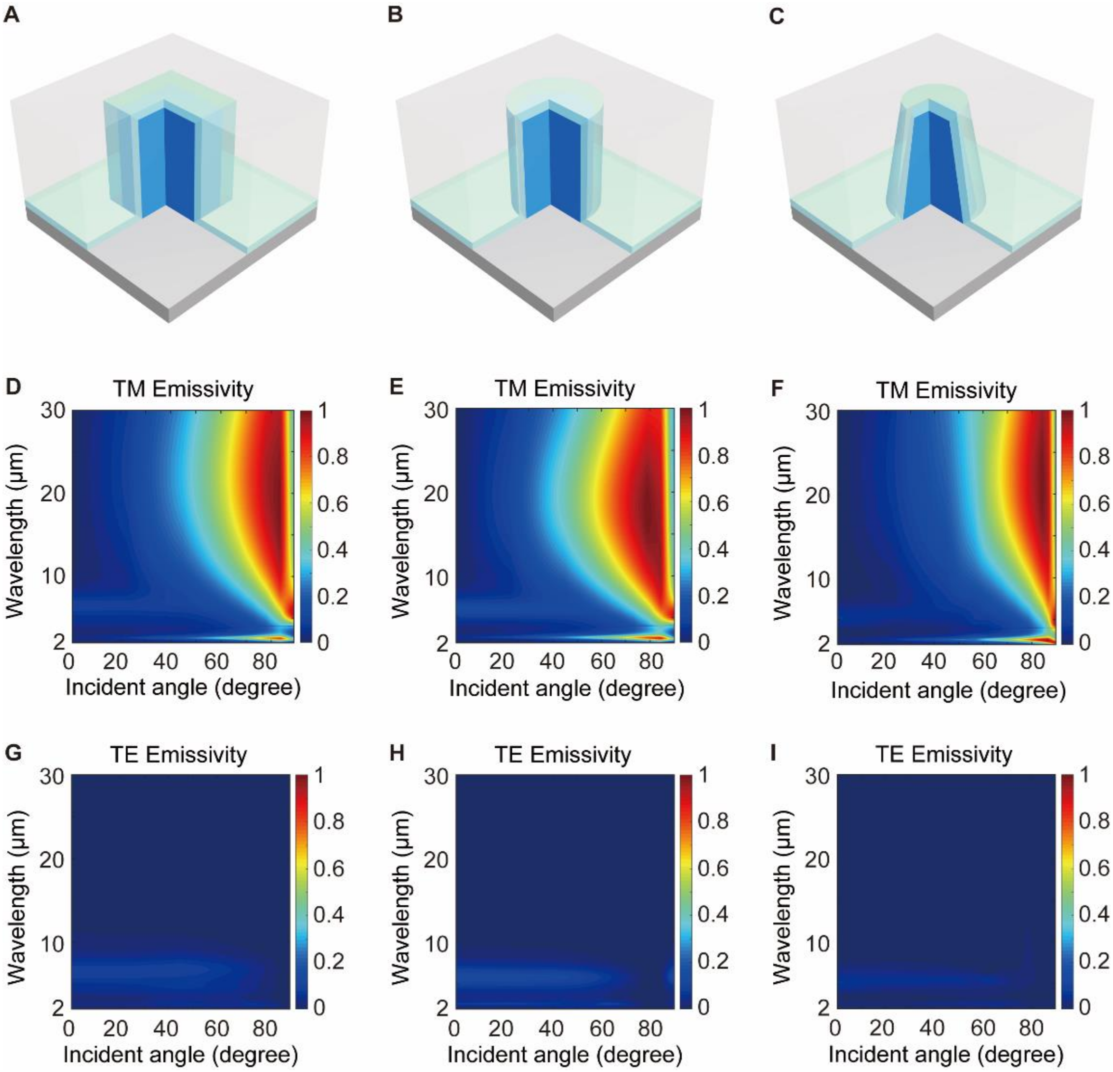

Figure S10. Robustness of the realistic design. (A-C) Schematic representations of the unit cells of the emitters based on three-phase composites with cuboid **(A)**, cylindrical **(B)**, and frustum-shaped **(C)** nanorods. **(D-F)** Full wave simulation of emissivity of the emitters based on three-phase composites corresponding to the three cases in A-C for the TM polarization. The same parameters: the volume fractions of materials, the height of the nanorods (1.5 μm), and the length of the unit cells (2 μm). **(G-I)** Full wave simulation of emissivity of the emitters corresponding to the three cases in D-F for the TE polarization ($\varepsilon_\infty = 3,\ \lambda_p = 1.5\ \mu m,\ \gamma = 0.1\ \omega_p,\ \varepsilon_2 = 1.5,\ \varepsilon_3 = 1$). It is confirmed that comparable outcomes can be achieved for the unit cell with different shapes but equal volume fractions and thicknesses. See Supplementary Note 8 for the detail parameters.

Supplementary Note 7: Polarization selectivity.

The polarization dependence of the Berreman mode has been demonstrated in previous work through interference theory [4] (within the ENZ regime, the same case as the two-phase metamaterials in this work). For the system shown in Figure S11A, the reflection coefficient (r) of the system can be written as:

$$r = \frac{r_{12}+r_{23}e^{2i\delta}}{1+r_{12}r_{23}e^{2i\delta}} = \frac{r_{12}-e^{2i\delta}}{1-r_{12}e^{2i\delta}} \quad \text{(S3)}$$

where r_{ij} is the Fresnel reflection coefficient for incidence from medium i to j, $\delta = k_{z2}d$, $k_{z2} = k_0\sqrt{\varepsilon_{am} - \varepsilon_{air}sin^2\theta}$ is the wave vector component normal to the interface in the thin film, ε_{am} is the permittivity of the absorbing medium (medium 2), k_0 is the free space wave number, and θ is the angle of incidence. For a PEC substrate, $r_{23} = -1$. For strong absorption (emission), two conditions need to be satisfied: $|r_{12}| \approx |e^{2i\delta}|$ and $\phi_{12} \approx \phi_d + 2m\pi$. ϕ_{12} is the phase of r_{12}; ϕ_d is the phase of $e^{2i\delta}$. Figure S11B plots ϕ_{12} and ϕ_d for the system shown in Figure 1E and 3C with d = 2 μm, at λ =11 μm (spectral position of E_{max} near unit), ε_{am} = 1.01 + 0.03i for the TM and TE polarization. According to these results, it is clear that TE polarization cannot satisfy both conditions. Therefore, the emitters proposed are polarization-dependent in these conditions.

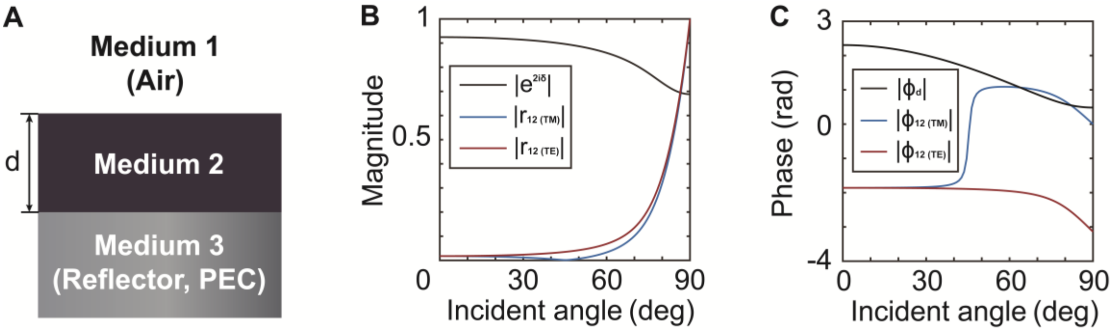

Figure S11: Numerical verification of the polarization dependence of three-phase metamaterial-based emitters. (A) Schematic of a typical directional thermal emitter consisting of medium 1,2, and 3. (B) Magnitude of $e^{2i\delta}$ and r_{12} for TM and TE polarization. **(C)** Phase of $e^{2i\delta}$ and r_{12} for TM and TE polarization.

To confirm the polarization-dependent behaviors, we perform full-wave simulations for the electric field distributions (averaged for the xz and yz cross sections) of the emitter based on the three-phase metamaterial as shown in Figure 3A-B at 11 μm with the incident angle equal to 84° (Figure S12) for the TE and TM polarizations. The wavelength and incidence angle correspond to

the spectral and angular position of E_{max}. Actually, for the emitter based on a three-phase absorbing medium (Figure 3A-B), the maximum electric field equal to 1.13×10^8 V/m (TM), approximately.

According to the full-wave simulation results, there is a strong electric field localization at the incidence of TM-polarized light, whereas there is no such effect for TE-polarization. Therefore, the emitters proposed are polarization-dependent.

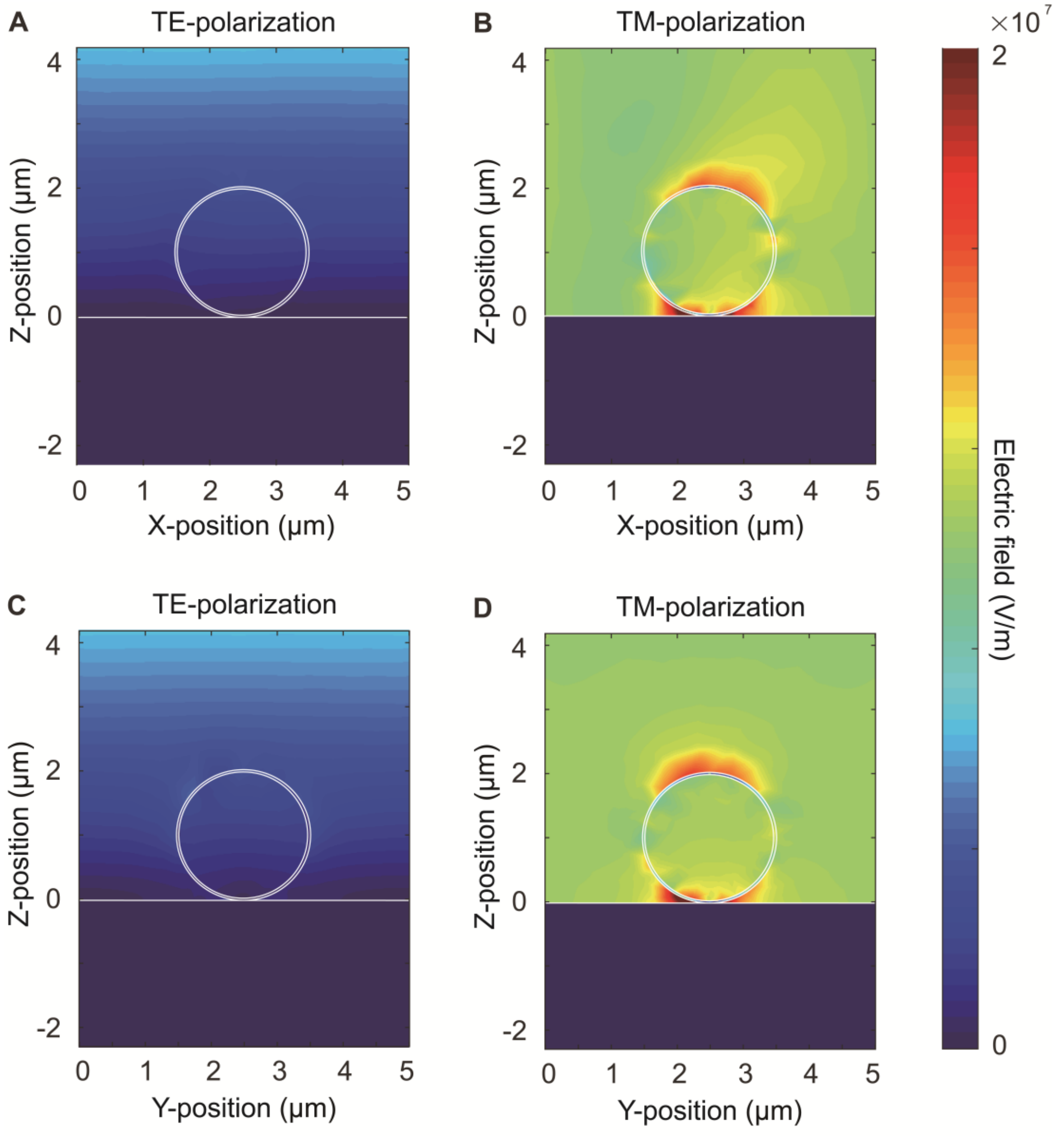

Figure S12: Full-wave simulations for the electric field distributions (averaged for the xz and yz cross sections) of the emitter based on the three-phase metamaterial as shown in Figure 3A-B at 11 μm with the incident angle equal to 84°.

Supplementary Note 8: Detailed unit cell parameters of all designed emitters.

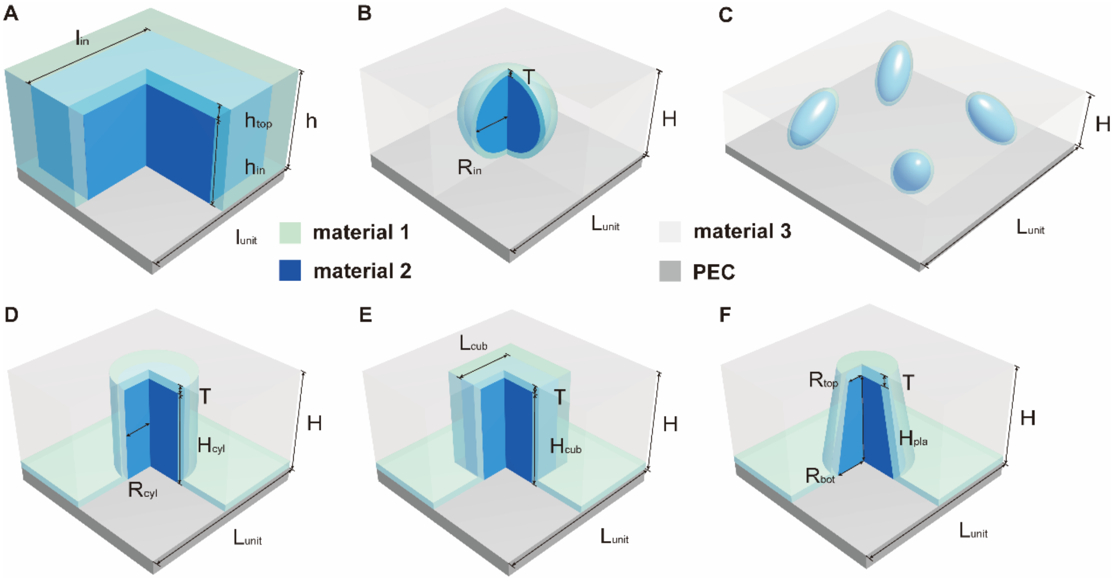

Figure S13. Schematic representations of the unit cells.

1. Two-Phase metamaterials (Figure S13A)

**Emissivity**	h_{top} (μm)	h_{in} (μm)	L_{in} (μm)	L_{unit} (μm)	
Figure 2E	0.01	0.39	0.48	0.5	
**Emissivity**	f_1/h_1 (μm)	f_2/h_2 (μm)	f_3/h_3 (μm)	f_4/h_4 (μm)	f_5/h_5 (μm)
Figure S2E	0.98/0.3	0.93/0.07	0.86/0.03	0.75/0.025	0.6/0.025
Figure S2F	0.955/0.1	0.6/0.03	0.45/0.022	0.2/0.022	

2. Three-phase metamaterials

<u>Figure S13B</u>

**Emissivity**	T (μm)	R_{in} (μm)	H (μm)	L_{unit} (μm)
Figure 1C	0.001	0.05	0.102	0.15
Figure 1D	0.003	0.2	0.406	0.8
Figure 1E	0.017	1	2.034	5
Figure 3D	0.017	1	2.034	5
Figure S5-6	0.01	0.5	1.02	2
Figure S7B	0.01	0.5	1.02	2
Figure S7C	0.01	0.5	1.02	4

Figure S7D	0.01	1	2.02	4
Figure S9C	0.015	0.75	1.53	5

Figure S13C

**Emissivity**	A_{lon} (μm)	A_{sho} (μm)	T (μm)	H (μm)	L_{unit} (μm)
Figure 4B	2	0.25	0.01	2	3

A_{lon}: The long axis length of the ellipsoid particle made of material 2.

A_{sho}: The short axis length of the ellipsoid particle made of material 2.

T: The thickness of the layer made of material 1.

Figure S13D

**Emissivity**	T (μm)	H_{cyl} (μm)	R_{cyl} (μm)	L_{unit} (μm)
Figure 4D	0.01	2	0.4	5
Figure S9F	0.01	2	0.3	3
Figure S10E, H	0.015	1.5	0.1	2

Figure S13E

**Emissivity**	T (μm)	H_{cub} (μm)	L_{cub} (μm)	L_{unit} (μm)
Figure S10D, G	0.015	1.5	0.15	2

Figure S13F

**Emissivity**	T (μm)	H_{cub} (μm)	R_{top} (μm)	R_{bot} (μm)	L_{unit} (μm)
Figure S10F, I	0.015	1.5	0.05	0.1	2

References

[1] V. A. Markel, “Introduction to the Maxwell Garnett approximation: tutorial,” J. Opt. Soc. Am. A, vol. 33, no. 7, pp. 1244, 2016.

[2] L. Sun, K. W. Yu, and G. P. Wang, “Design Anisotropic Broadband ϵ -Near-Zero Metamaterials: Rigorous Use of Bergman and Milton Spectral Representations,” Phys. Rev. Applied, vol. 9, no. 6, pp. 064020, 2018.

[3] S. B. Jones and S. P. Friedman, “Particle shape effects on the effective permittivity of anisotropic or isotropic media consisting of aligned or randomly oriented ellipsoidal particles,” Water Resour. Res., vol. 36, no. 10, pp. 2821–2833, 2000.

[4] B. Johns, S. Chattopadhyay, and J. Mitra, “Tailoring Infrared Absorption and Thermal Emission with Ultrathin Film Interferences in Epsilon-Near-Zero Media,” Adv. Photonics Res., vol. 3, no. 1, pp. 2100153, 2022.
